# Utilization of Eggshell Membrane and Olive Leaf Extract for the Preparation of Functional Materials

**DOI:** 10.3390/foods10040806

**Published:** 2021-04-09

**Authors:** Oguz Bayraktar, Charis M. Galanakis, Turki M. S. Aldawoud, Salam A. Ibrahim, Merve Deniz Köse, Mehmet Emin Uslu

**Affiliations:** 1 Department of Bioengineering, Ege University, 35100 Bornova-Izmir, Turkey; 2 Research & Innovation Department, Galanakis Laboratories, 73131 Chania, Greece; cgalanakis@chemlab.gr; 3 Food Waste Recovery Group, ISEKI Food Association, 1190 Vienna, Austria; 4 Department of Botany & Microbiology, College of Science, King Saud University, Riyadh 11451, Saudi Arabia; tdawoud@ksu.edu.sa; 5 Food and Nutritional Sciences Program, North Carolina A&T State University, Greensboro, NC 27411, USA; ibrah001@ncat.edu; 6 Department of Chemical Engineering, Ege University, 35100 Bornova-Izmir, Turkey; mervedenizkose@gmail.com; 7 Department of Bioengineering, Manisa Celal Bayar University, 45140 Manisa, Turkey; m.eminuslu@gmail.com

**Keywords:** eggshell membrane, olive leaf extract, functional material, antimicrobial, cytotoxicity

## Abstract

Eggshell membrane (ESM) is a natural proteinaceous by-product of the food industry, especially in the pasteurized egg industry, resulting in the availability of much discarded egg waste. In the literature, eggshell (ES) and ESM usage for their adsorbent properties to remove various organic and inorganic hazardous chemicals, especially from wastewater, has gained interest. In addition, agricultural (olive leaf) and food industry (eggshell and eggshell membrane) waste can together be valorized to produce value-added functional products. This study’s objective was to evaluate the eggshell membrane’s loading capacity for bioactive compounds obtained from olive leaf extract (OLE) in order to prepare functional biomaterial. In this study, waste eggshell membranes were used to adsorb the phenolic compounds from olive leaf extract to design functional biomaterials. Using the foam separation method, both separation of the eggshell membrane and adsorption of bioactive compounds to the eggshell membrane were achieved simultaneously. The characterization studies showed that OLE was successfully adsorbed to the eggshell membrane. Cytotoxicity and antimicrobial studies showed that prepared OLE-loaded membranes were functional materials with bioactive properties. In conclusion, ESM was determined as a promising protein in the production of functional antioxidative and antimicrobial food or dietary supplement after the adsorption of bioactive olive leaf polyphenols.

## 1. Introduction

The eggshell membrane (ESM) is a part of the egg that contains particular essential and widely used nutrients. The utility of ESM, together with eggshell (ES), has long been underestimated because it was considered waste material. Despite its fascinating structure and unique properties, most eggshell waste is discarded without further processing by being sent to a landfill, at a high cost per ton depending on the landfill location [1,2]. Due to valuable organic and inorganic components present in the eggshell, this waste can be utilized to prepare value-added functional products [3,4]. 

The economic burden of the eggshell waste disposal and the adsorption properties of the eggshell membrane have created significant interest in the utilization of the eggshell waste for the preparation of functional materials. The components of eggshell waste can be classified into two main groups: the inorganic part, comprising the calcified eggshell made of calcite and calcium carbonate crystals, and the organic part of the eggshell membrane [5]. Eggshell waste is one of the most abundant natural wastes. It is created in substantial, massive amounts by the pasteurized egg industry, in addition to the daily consumption of eggs [6,7]. The eggshell membrane’s organic part includes valuable biomaterials such as collagen, polypeptides, and amino acids [8].

The olive tree (*Olea europaea L.),* endemic to the Mediterranean countries, has been widely used in traditional medicine over decades. However, olive leaves are now considered as the waste product of olive farming and processing. The production of olive oil and table olives generates many olive leaves, which can be utilized to obtain natural products with bioactive compounds as food additives, functional food, and nutraceutical products. Recently, various beneficial health properties, such as the antioxidant effect, anti-hypertensive activity, hypoglycemic effect, and anti-inflammatory properties, of olive leaves and their extract have been studied. In the literature, there are studies for the antimicrobial activities of olive leaf extract (OLE) against bacteria, fungi, and viruses [9,10,11].

Besides being an economic burden for producers, olive oil by-products also represent a severe environmental problem [12]. Simultaneously, these are rich in bioactive compounds, which are remarkable added-value ingredients for other industries (e.g., for the meat sector or cosmetics [13,14,15]). In the literature, components of the olive leaf extract were investigated. The most abundant bioactive phenolic constituent in olive leaf is oleuropein, 6–9% of dry olive leaf [16]. Hydroxytyrosol, verbascoside, luteolin, diosmetin, rutin, luteolin-7-glucoside, and apigenin-7-glucoside are the other phenolic compounds present in the olive leaf extract [17,18].

Nowadays, there has been a growing interest in the development and commercialization of functional foods, nutraceuticals, and dietary supplements from natural sources to promote health benefits [19]. For this purpose, the valorization of food processing by-products [20,21] and particularly those obtained from the olive processing sector [22] as cheap and abundant sources of bioactive components have attracted the researcher’s interest. Olive leaves are increasingly important as an herbal remedy, and they are commercialized in the pharmaceutical market at premium prices as intact leaves or extracts. For instance, following the catastrophic effects of the COVID-19 pandemic, immunity is among consumers’ highest priorities, who are interested in increasing the consumption of functional foods in their diets [23,24,25]. 

The main objective of this study was to valorize two waste materials (eggshell membranes and olive leaves) to prepare value-added functional food. Therefore, this study aimed to separate the eggshell membrane from the eggshells that constitute an important waste problem in the food sector, and to enrich the composition with natural compounds having high antioxidant and antimicrobial properties that shall be added during the foam separation process. As a result, the waste that is abundant following the production of pasteurized liquid eggs and olive leaves, which is also qualified as food and agricultural waste, can also be utilized. Moreover, the addition of extra functionality through adsorption by using the hydrophobic interaction property of the eggshell membranes, and the addition of OLE that is rich in antioxidants, helps to enrich the functionality of the ESM. By using ESM with multifunctional bioactivities in the food and cosmetics industry, a serious contribution can be made to reduce cost rates.

## 2. Materials and Method

### 2.1. Materials

Eggshell membranes were obtained from commercial eggshell waste. Eggshell waste was washed with ozonated water for removing the microbial load on it. Acetic acid (glacial 100% anhydrous, Merck, Darmstadt, Germany) and sodium hydroxide (for analysis, Merck, Darmstadt, Germany) were purchased. Olive leaves were collected from Urla, İzmir region. Oleuropein, hydroxytyrosol, and verbascoside (purity by HPLC, ≥98%) were purchased from Sigma Aldrich (St. Louis, MO, USA). All of the other chemicals used were of analytical grade. Acetonitrile (Chromasolv gradient grade for HPLC), acetic acid, ethanol, and hydrochloric acid were from Sigma-Aldrich, St. Louis, MO, USA. All solutions were prepared with distilled water.

### 2.2. Methods

#### 2.2.1. Preparation of Olive Leaf Extract

Olive leaf extract was prepared as given in our previous study. The solid-to-liquid ratio was determined from that study [26]. The collected olive leaves were washed with deionized water before being dried at 37 °C. Then ground olive leaves were mixed with 70% aqueous ethanol solution with a 1:20 solid-to-liquid ratio. Extraction was carried out at 35 °C for two hours at 180 rpm. After two hours, filtration was done to remove the insoluble solids. Then ethanol in the remaining solution was removed with a rotary evaporator. The obtained aqueous solution was freeze-dried to obtain olive leaf extract (OLE) as a dried powder. 

#### 2.2.2. Separation of Eggshell Membrane from Eggshell Waste and Preparation of OLE-Loaded ESM

**Figure 1:**
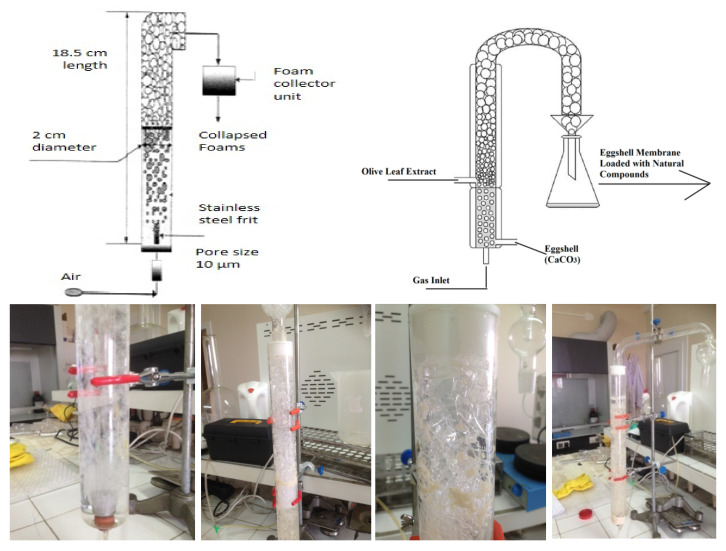
Experimental set-up used for the preparation of OLE (olive leaf extract)-loaded eggshell membrane.

The foam separator separates the bioactive compounds from the solution using their hydrophilic and hydrophobic properties. When air is given to the solution, hydrophobic compounds are attached to the foam formed, and hydrophilic substances remain in the aqueous solution. When the rising foam is collected in a separate container, the hydrophobic materials will be collected. By using the foam separator set-up shown in Figure 1, both separations of ESM from eggshell waste and adsorption of olive leaf polyphenols on the separated eggshell membrane were achieved simultaneously. Solutions at various concentrations of acetic acid were introduced to the system to determine the optimum conditions for the desired process responses. After the optimum separation conditions were determined, OLE-loaded eggshell membranes were fabricated in the foam separator. Prepared OLE was introduced to the system with airflow rate.

Separation efficiency percentages of the membrane and shell were calculated by considering the ratio of the amount of eggshell membrane to the amount of material (eggshell membrane + eggshell) at the top of the foam collector.

#### 2.2.3. Experimental Design

Effects of acetic acid concentration, average eggshell size, airflow rate, and treatment time in the column were investigated with Central Composite Design (CCD) in response surface methodology (RSM) by Minitab Version 7.0 (Minitab, LLC, State College, PA, USA). The separation efficiency of the eggshell membrane (%) was the main response chosen to determine the optimum process parameters. In Table 1, independent variables and their levels are given for the experiments, in order to investigate the separation efficiency of ESM from eggshell waste material. In order to load the olive leaf extract on ESM while separating the eggshell, a different set of experiments was performed. The factors in the experimental design used in the preparation of OLE loaded into the ESM in the foam separator were determined as follows. Effects of solid-to-liquid ratio (X1), treatment time in the column (X2), and ethanol concentration (X3) were investigated (Table S2, Supplementary Data). In these preliminary experiments, in order to determine the most suitable OLE, the sample with high-level results in two parameters was selected. These parameters are total phenol content and total antioxidant capacity. The total antioxidant capacity and total phenol content of the ESM loaded with OLE were the main responses chosen to determine the optimum process parameters. 

#### 2.2.4. Characterization of Eggshell Membrane and OLE-Loaded Eggshell Membrane

##### Fourier Transform Infrared (FTIR) Spectroscopy Analysis

Characterization of ESM and its constituents and OLE-loaded ESM were done with FTIR analysis. The FTIR spectra of the individual samples were recorded in 650–4000 cm^−1^ using Perkin Elmer Spectrum 100 FTIR spectrometer, Buckinghamshire, UK).

##### Scanning Electron Microscopy (SEM) Energy-Dispersive X-ray Spectroscopy (EDS) Analysis

To further investigate the changes in ESM morphologies, SEM analysis was performed with Philips XL 30S FEG, Amsterdam, Netherlands before and after loading OLE polyphenols on ESM. Elemental analysis of the eggshell membrane and OLE-loaded eggshell membrane was performed with an SEM-EDX detector. Samples were coated with a gold layer under argon gas before the SEM analysis.

##### HPLC Analyses

The chromatographic separation was carried out using a mobile gradient phase, 2.5% acetic acid-water solution (A) and 100% acetonitrile (B) as a solvent with a flow-rate of 1.0 mL/min at 30 °C. The analytical column was C18 LiChrospher 100 5 µm (4 × 250 mm) (Sigma Aldrich, USA). The analyses were carried out with UltiMate 3000 (Thermo Scientific, Waltham, MA, USA). The detection of the compounds was established at 280 nm. The chromatographic separation was carried out using mobile phase A acetic acid-water (2.5:97.5, v/v) and mobile phase B acetonitrile with the gradient program: initially with 95% A to 5% B; 0–20 min linear gradient to 25% B; 20–40 min linear gradient to 50% B; 40–50 min linear gradient to 80% B; 50–60 min linear gradient to 95% B. The standard solutions of OLE polyphenols were prepared in the mobile phase.

#### 2.2.5. Determination of Total Antioxidant Capacity (TEAC) and Total Phenol Content (TPC) for OLE

The antioxidant capacities of the samples were determined with the ABTS method. ABTS+ (ABTS radical cation) method was used with Trolox as a standard antioxidant for the antioxidant analysis. As stated in the literature, 7 mM aqueous ABTS solution was mixed with 2.45 mM potassium persulfate solution to form ABTS+. The prepared mixture was then left in the dark at ambient temperature for 12–16 h to complete the reaction. The ABTS+ solution was diluted with ethanol to obtain 0.7 A (±0.02) at 734 nm. Each sample was analyzed 3 times. Antioxidant capacities of the samples were calculated as Trolox equivalent. Sample (10 µL) and ABTS solution (200 µL) were mixed and waited in the dark for 30 min. Then absorbance values were recorded at 734 nm with a spectrophotometer (Varioskan, Thermo Fischer, Waltham, MA, USA).

Total phenolic content was determined with the Folin-Ciocalteu method using gallic acid as a standard. Samples were prepared as 20 μL of the diluted sample solution and 100 μL Folin-Ciocalteu reagent previously diluted with a 1:10 ratio in a 96-well plate. Then 80 µL 7.5% Na_2_CO_3_ solution was added to the mixture and waited in the dark for an hour. Then absorbance values were recorded at 725 nm with a spectrophotometer (Varioskan, Thermo Fischer, USA).

#### 2.2.6. Determination of Cytotoxic Activity for OLE-Loaded ESM (MTT Method)

The cytotoxic effect of olive leaf extracts on 3T3 mouse fibroblast cells was determined by the MTT method. In the first step of the method, fibroblast cells were seeded in 96-well plates. Cells were separated from flasks using trypsin-EDTA and counted after centrifugation. Cells were seeded into the wells in a volume of 95 µL as 10,000 cells/well. Antibiotics were not used in the DMEM content, which is the cell culture medium, against the risk of affecting the analysis results. Cells cultured in a 96-well plate were incubated for 24 h at 37 °C, 5% CO_2_, and the cells were allowed to attach to the well bases. On the second day of the procedure, lyophilized olive leaf extract was dissolved in the antibiotic-free medium by vortexing, and sterilized by filtering with a 0.22 µm injector. Prepared samples were subjected to UV sterilization for 2 h: 1 h on one surface, and 1 h on the other. Working concentrations were calculated to be 20 times higher, since 5 µL of the extract was added on 95 µL of cell suspension in each well, and the extract concentration would decrease 20 times. In order to observe the effects of the extract more comprehensively, the concentration range was chosen as 100–3000 µg/mL. To each well, 5 µL of the extract was added on the cells attached to the plate surface. An extract-free cell suspension was used as a negative control, while each concentration and control set were prepared in triplicate. Cells were incubated at 37 °C, 5% CO_2_, at intervals of 24–48 and 72 h.

On the third day, the analysis day, the MTT solution was prepared to be added to the first experiment set, the 24-h experiment group. Powdered MTT was dissolved in 1×PBS buffer solution at a stock concentration of 5 mg/mL. The set of experiments to be analyzed was removed from the incubator. The extract solution mixed with the cell culture medium in the wells was removed by inverting the plate, before 100 µL of 1×PBS isotonic solution was added to the cells adhered to the plate surface, and the culture medium residues were removed. The amount of MTT solution to be used for each experiment set was diluted to its working concentration of 0.5 mg/mL, and 100 µL of 0.5 mg/mL MTT solution was added to the wells containing cells and blank wells reserved for spectrophotometric analysis. The prepared plate was wrapped in aluminum foil and incubated at 37 °C in the dark for 4 h. At the end of the incubation period, the plate was centrifuged at 1800 rpm for 10 min. The formazan crystals formed due to the reduction of MTT by the mitochondrial reductase enzyme in living cells became pellets at the bottom of the plate. After removing the supernatant plate by inversion, the formazan crystals were dissolved with DMSO, and 100 µL of pure DMSO was added to each well. The plate was then vortexed at approximately 400–500 rpm for 5 min. The 96-well plate was read at 545 nm wavelength, and absorbance values corresponding to the concentrations were obtained. The mean absorbance values of the replicas were averaged, and standard deviation values were calculated. Cell viability was calculated with the given Equation 1.

Viability % = (Sample Absorbance Value/Control Absorbance) × 100(1)

#### 2.2.7. Determination of Antimicrobial Activity of OLE-Loaded ESM with Agar Method

For the determination of the eggshell membrane’s antimicrobial activity (as used in the cytotoxic activity), membranes cut to a surface area of 6 cm^2^ were placed in 3 mL of medium in 12-well plates. The membranes were incubated for 24 h at 37 °C and 70 rpm shaking condition after seeding, and there were calculated to be 10^6^ colonies of *E. coli* in 3 mL of the medium at the final concentration. It was inoculated on agar at the end of 24 h with 10^6^ dilutions and incubated at 37 °C for 24 h for colony counting.

## 3. Results and Discussion

### 3.1. Characterization of Eggshell Waste and Eggshell Membrane

The biggest challenge faced with discarded commercial eggshell waste is the microbial load. To overcome this problem, washing the eggshell wastes with ozonated water as a pretreatment was carried out. With ozone pretreatment, the initial microbial load of the eggshell waste was decreased from 1,500,000–2,000,000 cfu/g to 1000–10,000 cfu/g. For the effective separation of the eggshell membrane during the foam separation process, particle size analysis of eggshell waste was performed as the histogram, relative percentage distribution, and cumulative percentage distribution. As a result of this analysis, it was found that the particle size varied in the range of 250–2000 microns, and the average particle size (30–35% of the total amount) was about 500 microns. However, this particle size can further be reduced with the grinding process. Different average eggshell waste sizes were chosen to be used in the foam separation set-up to separate the eggshell membrane from the eggshell.

**Figure 2:**
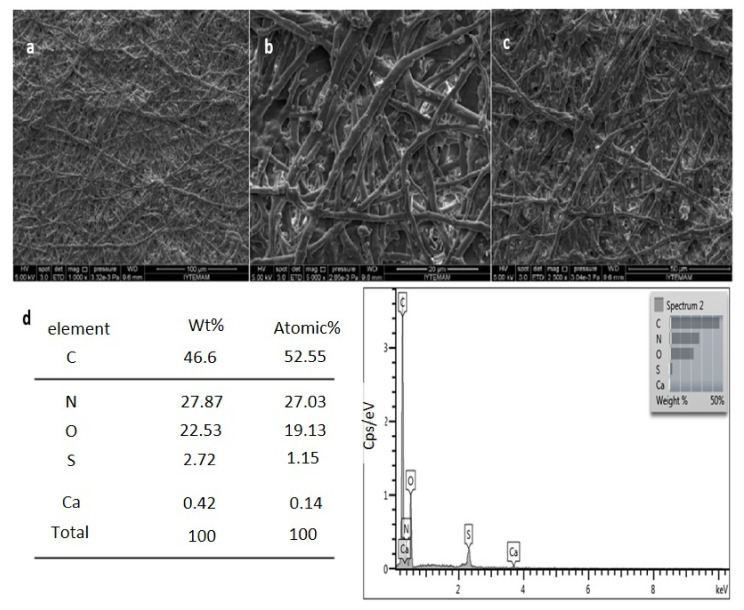
SEM images of the eggshell membrane at (**a**) 1000×, (**b**) 5000×, (**c**) 2500×; (**d**) EDS analysis of eggshell membrane.

In Figure 2, SEM images and EDS analyses of the eggshell membrane are given.

SEM images revealed the porous and fibrous structure of the eggshell membrane, as seen in Figure 2a–c. The EDS analysis showed that the eggshell membrane consisted of C, N, O, S, and Ca, with 46.46, 27.87, 22.53, 2.72, and 0.42 wt%, respectively. Ca found in the membrane was due to the presence the CaCO_3_ in the eggshell waste. When the percentage distributions of the elements were examined, carbon, nitrogen, and oxygen elements were detected in high amounts due to proteins, collagen, and glycoproteins from the organic matrix. S was detected due to the disulfide bonds in the structure of amino acids. All these element distribution percentages are compatible with the eggshell membrane structure and comply with the literature [7]. ESM has mainly 80–85% organic matrix (as primary structure (10%) collagen type I, V and X as secondary structure (70–75%) osteopontin, fibronectin, glycoproteins, and proteoglycans) and 15–20% inorganic matrix (CaCO_3_). Therefore, eggshell membrane and eggshell have gained interest as a source for food and health applications [7]. A protein-rich eggshell membrane could be used as a coating for fat uptake reduction in deep-fat fried meat products [27].

**Figure 3:**
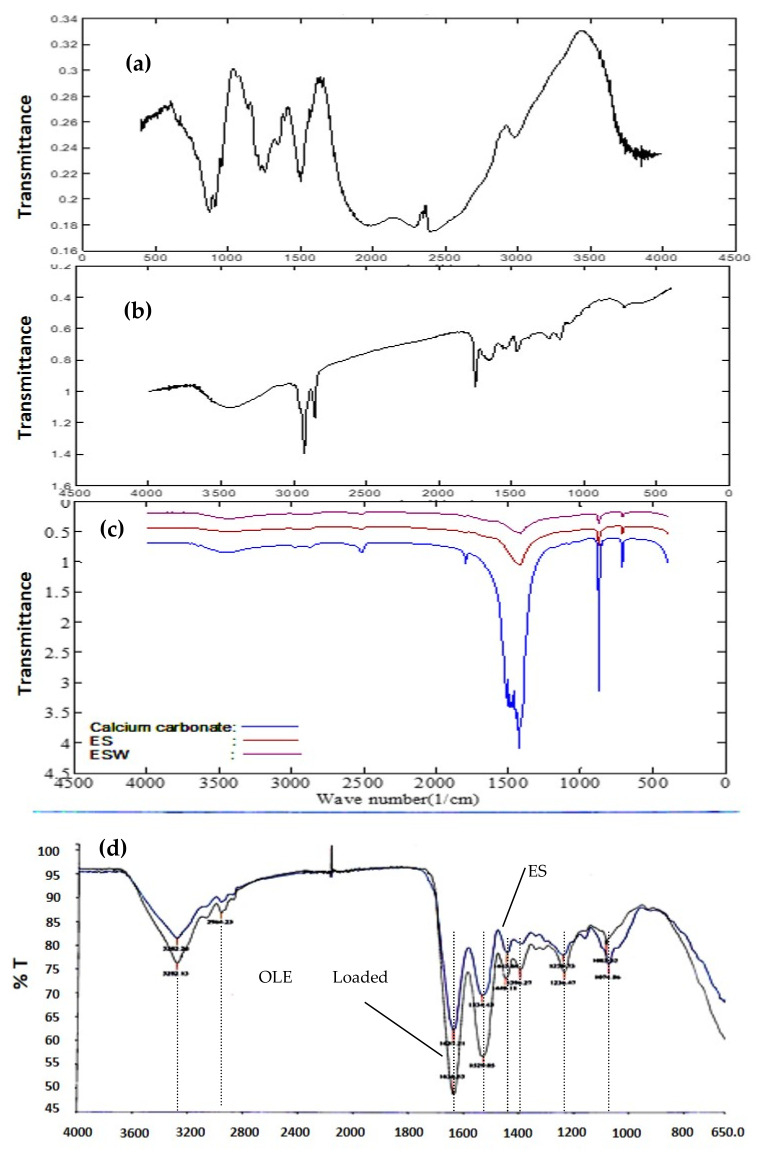
FTIR spectra of the (**a**) hyaluronic acid; (**b**) collagen; (**c**) eggshell waste (ESW), eggshell (ES), and calcium carbonate; and (**d**) eggshell membrane (ESM) and olive leaf extract (OLE)-loaded eggshell membrane (ESM).

In Figure 3, FTIR spectra of the components of the eggshell and eggshell membrane are given. 

The FTIR spectrum of the components present in the eggshell membrane is given in Figure 3a,b. In Figure 3c, FTIR spectra of calcium carbonate, eggshell waste, and eggshell membrane are shown. Two sharp, distinct bands observed at 1650 cm^−1^ and 1440 cm^−1^ were identified as amide I (C=O stretching vibrations) and amide II (CN stretching and NH in-plane bending), respectively [28,29].

The highest intensity peak was observed at 1417 cm^−1^, which is strongly associated with the presence of carbonate minerals within the eggshell matrix. The other two peaks observed at 712 cm^−1^ and 875 cm^−1^ are associated with the in-plane deformation and out-plane deformation modes, respectively, in the presence of calcium carbonate. On the other hand, the ESM sample showed significant peaks at the intensities of 3200–3500, 1651, 1538, and 1384 cm^−1^, which are associated with amines and amides [30]. As seen from our FTIR spectrum given in Figure 3d, these results are in accordance with the FTIR results reported in the literature. All these structural properties and bonds are compatible with existing mineral and biopolymer structural properties present in ESW, ES, and ESM.

**Figure 4:**
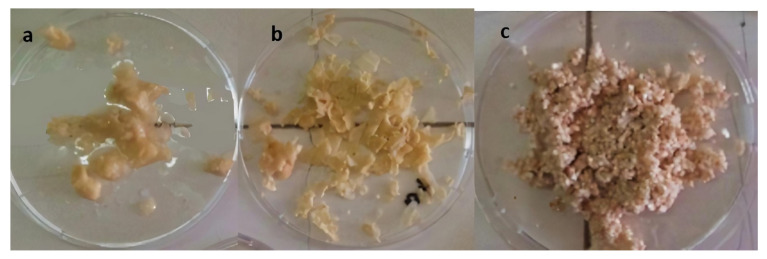
Images of eggshell membrane (**a**,**b**) and eggshell (**c**) obtained at the top and bottom of the foam separator, respectively.

Figure 4 shows images of the separated eggshell and eggshell membrane after the foam separation process.

In the foam separator, the eggshell and the membrane were obtained from the column’s bottom and upper parts, respectively (Figure 4). The eggshell and membrane can be separated with sufficiently good efficiency in these operating conditions. Then, the eggshell and eggshell membrane appearing in Figure 4 were dried, their weight was measured, and the separation efficiency percentages of the membrane and shell were calculated. The separation efficiency of the eggshell and eggshell membrane was dependent on the foam separation process parameters. The results revealed that 90% of the initially added eggshell waste was obtained from the process. Only 10% of the eggshell waste was lost during the process. The separation efficiency of the eggshell and eggshell membrane from the eggshell waste was found as 81% ± 4. The yield for the recovery of the eggshell membrane and eggshell from eggshell waste was found as 6% ± 3 and 83% ± 4, respectively.

### 3.2. Characterization of Olive Leaf Extract

Based on our previous studies [31,32], to achieve the highest total phenol content, olive leaf extraction was done with 70% aqueous ethanol solution. The total phenolic content of the OLE was determined as 10 mg GA/g olive leaf extract.

**Figure 5:**
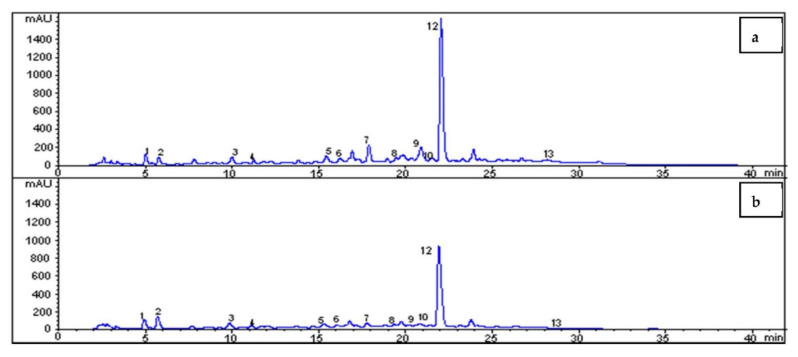
HPLC Chromatogram of addition of OLE in ESM at 2 different concentrations: (**a**) 3% (*w/v*) and (**b**) 2% (*w/v*). (1) hydroxytyrosol, (2) tyrosol, (3) catechin, (4) caffeic acid, (5) vanillic acid, (6) vanillin, (7) rutin, (8) luteolin-7-glucoside, (9) verbascoside, (10) apigenin-7-glucoside, (11) diosmetin-7-glucoside, (12) oleuropein, and (13) luteolin

The HPLC chromatogram of the OLE showed that oleuropein and rutin are the most abundant phenolic compounds in the prepared olive leaf extract (Figure 5). In addition, a total of 13 peaks were observed, as seen in the chromatogram. These were (1) hydroxytyrosol, (2) tyrosol, (3) catechin, (4) caffeic acid, (5) vanillic acid, (6) vanillin, (7) rutin, (8) luteolin-7-glucoside, (9) verbascoside, (10) apigenin-7-glucoside, (11) diosmetin-7-glucoside, (12) oleuropein, and (13) luteolin. In 1 g OLE, 134 mg oleuropein and 18 mg rutin were determined. The phenolic constituents of the prepared olive leaf extract shown in the HPLC chromatogram have significant bioactivities. As seen from Figure 5, with increasing OLE concentration (2% (*w/v*) and 3% (*w/v*)), peaks obtained from each compound were increased. The obtained data are in accordance with the findings reported in the literature. As stated in the literature, hydroxytyrosol, verbascoside, rutin, and oleuropein was present in the olive leaf extract [33]. When OLE is combined with biopolymers, ESM in our case, it can be widely used in many functional foods and biomedical applications [32,34].

#### 3.2.1. Response Surface Methodology (RSM) for the Separation of Eggshell Membrane

The experimental CCD design and obtained responses are given in Table 2. As seen from this table, the separation efficiency percentages of the membrane and shell varied between 34% and 81%.

The data given in Table 2 were examined with the analysis of variance, and the obtained results are given in Table S1 (Supplementary Data).

As seen in Table S1 (Supplementary Data), the obtained R^2^ value of 0.94 showed the experimental design’s validity. A relatively high coefficient of determination of the model (R^2^) of 0.94 indicated that the fitted second-order polynomial model accounted for 94% of the variance in the experimental data. There was no lack of fit. In the experimental design, the acetic acid concentration was the primary function. Values of *p* less than 0.05 indicate model terms are significant. In this case, A and A^2^ are significant model terms. According to the results of the statistical analysis, Equation (2) was obtained for separation efficiency %. The equation could be used to make predictions about the separation efficiency regarding acetic acid concentration.

Separation Efficiency (%) = 52.5707 − 0.1656 × A + 0.0036 × A^2^ (2)

If the R^2^ value was taken as 0.92, then in addition to the acetic acid concentration, treatment time in the column was also effective and useful for the prediction of the separation efficiency. After involving significant terms, the regression model for separation efficiency can be represented with Equation (3).

Separation Efficiency (%) = 52.5707 − 0.1656 × A + 0.0036 × A^2^ − 8.5237 × C + 1.4050 × C^2^ (3)

**Figure 6:**
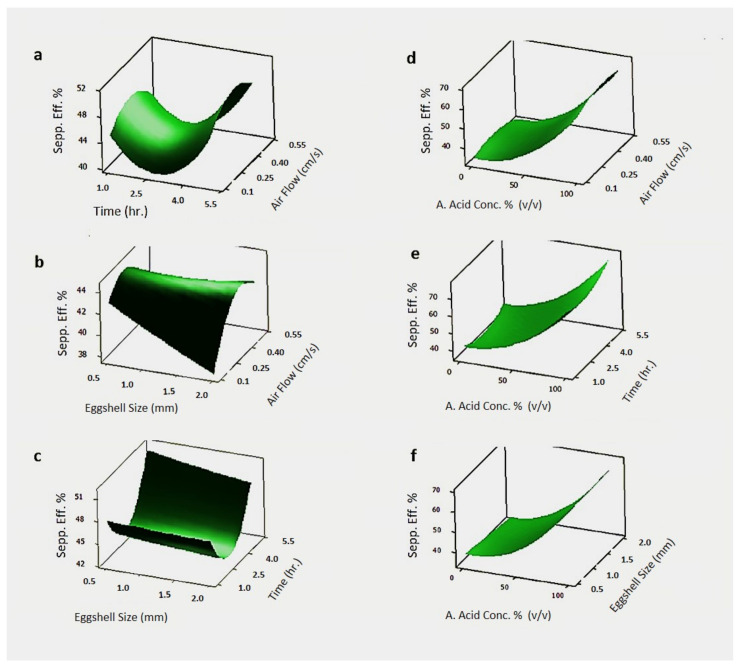
Response surface graphs of the separation efficiency (%) with acetic acid concentration 50%; (**a**) treatment time vs air flowrate (average eggshell size is 1.25 mm); (**b**) eggshell size vs air flowrate (treatment time 3 h); (**c**) eggshell size vs treatment time (air flowrate 0.3 cm/s); (**d**) acetic acid concentration vs air flowrate (average eggshell size 1.25 mm and treatment time 3 h); (**e**) acetic acid concentration vs treatment time (average eggshell size 1.25 mm and airflow rate 0.3 cm/h); (**f**) acetic acid concentration vs average eggshell size (treatment time 3 h and air flowrate 0.3 cm/s).

In Figure 6, the 3D response surface graphs of the experimental design with various process parameters are given.

In Figure 6a, the effect of the treatment time in the column and the airflow rate on the efficiency of membrane separation from the shell was examined. It was seen that the most efficient separation was achieved when the airflow rate was between 0.25 and 0.4 cm/s and the treatment time in the column was 5 h or more. Obtained data showed that the treatment time of 1 h in the column was sufficient for the achievement of reasonable separation efficiency. However, it has been observed that the separation efficiency values were enhanced with the prolongation to the higher contact time. This can be attributed to the higher contact time between acidic solution and eggshell waste, which results in weakened interaction between eggshell and eggshell membrane, as acetic acid concentration and shell particle size were kept constant at 50% and 1.25 mm, respectively. In Figure 6b, the effects of the shell size and airflow rate were examined on the efficiency of the membrane shell separation. The result showed that the most efficient separation was achieved in the case wherein the airflow rate was 0.4 cm/s, and the shell size was below 1 mm. The relatively higher separation efficiency can be obtained due to the smaller shell particle size with the higher surface area available for the contact of the acidic solution. However, shell particle size was not as significant as other parameters on the separation efficiency. Figure 6c shows that the effect of shell size and treatment time in the column together on the efficiency of membrane shell separation. It was observed that the shell size was not as effective as the other parameters. As clearly seen, the separation efficiency increased when the treatment time in the column was over 5 h.

In Figure 6, 3D response surface graphs of the experimental design with the various process parameters are also given. In Figure 6d–f, the effect of acetic acid % along with other effective process parameters are investigated. In Figure 6d, the effect of acetic acid concentration and airflow rate on the efficiency of membrane shell separation can be seen, and the graph shows that maximum separation efficiency was achieved when the acetic acid concentration was close to 100%, and the airflow rate in the column was around 0.4 cm/s. In Figure 6e, the effect of acetic acid concentration and treatment time in the column on the efficiency of membrane shell separation can be seen; separation efficiency increased when the acetic acid concentration was close to 100% and the treatment time in the column was around 4 h. For this analysis, the shell size and airflow rate were kept constant at 1.25 mm and 0.3 cm/s, respectively. In Figure 6f, the effect of acetic acid concentration and shell size on the efficiency of membrane shell separation is shown; it can be seen that the efficiency was increased when the acetic acid concentration was again close to 100%, while the shell size was not that effective on the separation efficiency values. The symmetrical distribution in the residual plots for antioxidant capacity shown in Figure S1a (Supplementary Data) shows the accuracy of the analysis.

#### 3.2.2. Effect of Process Parameters on the Total Phenolic Content and Antioxidant Capacity

In this study, the effects of process parameters on the total phenolic content and antioxidant capacity of the eggshell membrane due to the loaded olive leaf extract were investigated. As a result of the analysis, the ANOVA tables were obtained, and are given in Tables S3 and S4 (Supplementary Data).

The Model F-value implies the model is significant (Table S3 (Supplementary Data). Values of *p* less than 0.05 indicate model terms are significant. In this case, X2, X3, X2^2^, and X3^2^ are significant model terms. The coefficient of determination of the model (R^2^) was 0.96, which indicated a good fit between predicted values and the experimental data points (Table S3 (Supplementary Data)). After discarding insignificant terms, the regression model for total antioxidant capacity can be simplified to the following form in Equation (4).

Total Antiox.Capacity = 9312.7 + 490.16 × X2 − 119.77 × X3 − 21.51 × X1^2^ + 1.93 × X3^2^ (4)

**Figure 7:**
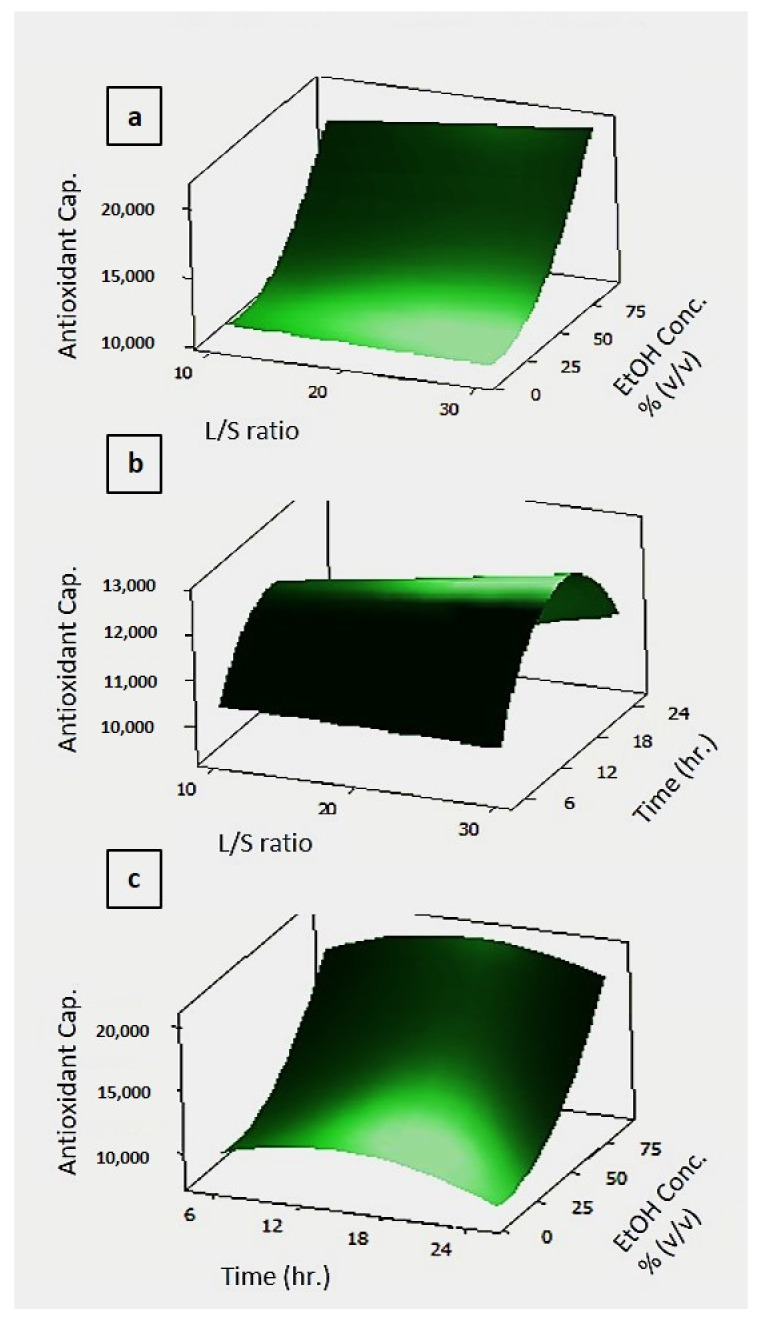
Effect of process parameters on the antioxidant capacity. In the figure, (**a**) antioxidant capacity vs EtOH concentration and L/S Ratio; (**b**) antioxidant capacity vs time and L/S Ratio; (**c**) antioxidant capacity vs EtOH concentration and time. The hold values are set for treatment time 15 h, ethanol concentration 50%, and L/S ratio 20. The unit for antioxidant capacity is μM TEAC (Trolox equivalent antioxidant capacity).

In Figure 7, 3D response surface graphs of the experimental design on antioxidant capacity with the various process parameters are given.

In Figure 7a, the effects of the EtOH concentration and L/S ratio were examined on the antioxidant capacity. It was observed that the highest antioxidant capacity was reached in the case where the EtOH concentration was nearly 75%, and the L/S ratio was not as effective as EtOH concentration. In Figure 7b, the dual effects of L/S ratio and time on antioxidant capacity were investigated by keeping ethanol concentration constant at 50%. It was determined that the highest antioxidant capacity was reached when the solid/liquid ratio approached 30, and the time was between 12 and 18 h. Figure 7c shows the effect of ethanol concentration and time on antioxidant capacity by keeping the L/S ratio at 20. The increase in antioxidant capacity when the treatment time was between 12 and 18 h and the ethanol concentration was 70% was confirmed by the other two graphs. The symmetrical distribution in the residual plots for antioxidant capacity shown in Figure S1b (Supplementary Data) shows the accuracy of the analysis.

**Figure 8:**
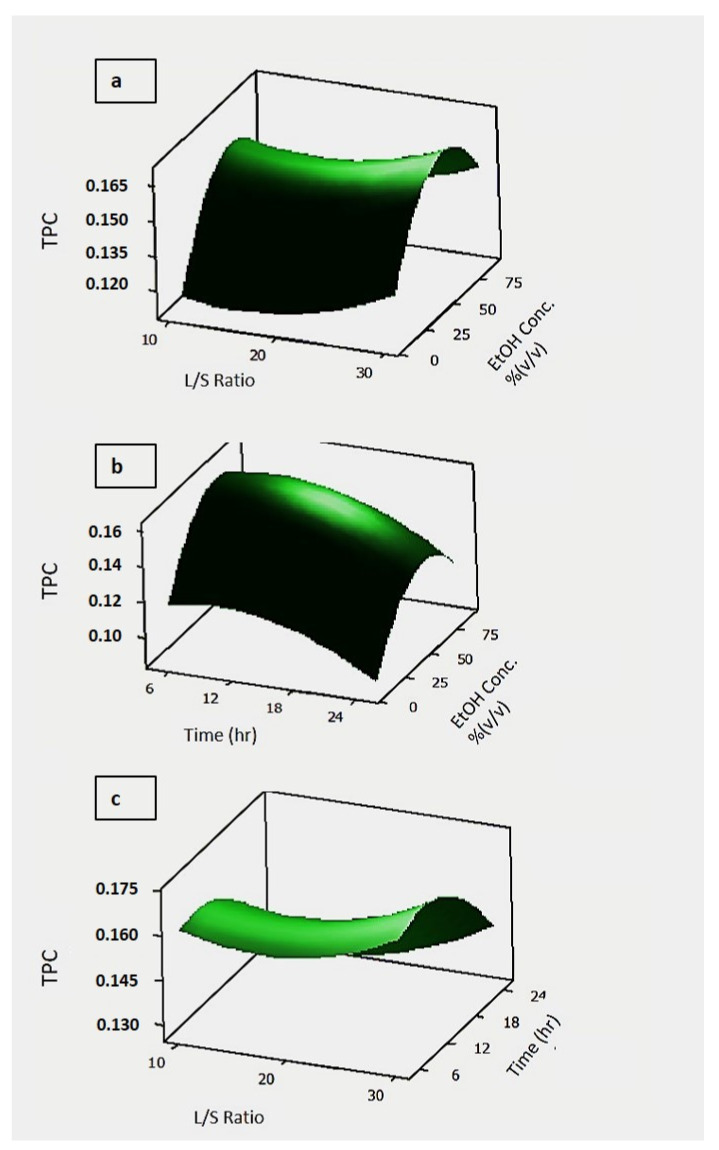
Effect of process parameters on the total phenol content. In the figure, (**a**) total phenolic content vs EtOH concentration and L/S Ratio; (**b**) total phenolic content vs time and EtOH concentration; (**c**) total phenolic content vs L/S ratio and time. The hold values are set for treatment time 15 h, ethanol concentration 50%, and L/S ratio 20. The unit for total phenolic content is mg GAE (gallic acid equivalent).

In Figure 8, 3D response surface graphs of the experimental design on total phenolic content with the various process parameters are given.

Figure 8a, the dual effects of solid/liquid ratio and time on total phenolic content capacity were investigated by keeping ethanol concentration constant at 50%. It was determined that the highest antioxidant capacity was reached when the solid/liquid ratio approached 30, and the time was between 12–18 h. Figure 8b showed the effect of ethanol concentration and time on total phenolic content. It was determined that the total amount of phenolic content increased when the time was kept for 6 h, and the ethanol concentration around 50%. When Figure 8c was examined, it was seen that the solid/liquid ratio and time did not show a very large substantial dual effect on total phenol. The symmetrical distribution in the residual plots for total phenolic content shown in Figure S1c (Supplementary Data) show the accuracy of the analysis.

As a result of the analysis, the ANOVA table was created and given in Table S4 (Supplementary Data).

The Model F-value implies the model is relatively significant (Table S4, Supplementary Data). Values of *p* less than 0.05 indicate model terms are significant. In this case, X3 and X3^2^ are significant model terms (Table S4, Supplementary Data). The coefficient of determination of the model (R^2^) was 0.72, which indicates a relatively good fit between predicted values and the experimental data points. After discarding insignificant terms, the regression model for total phenol content can be simplified to the following form in Equation (5).

Total Phenol Content = 0.105738 + 0.002304 × X3 − 0.000019 × X3^2^ (5)

### 3.3. Characterization of OLE-Loaded ESM

**Figure 9:**
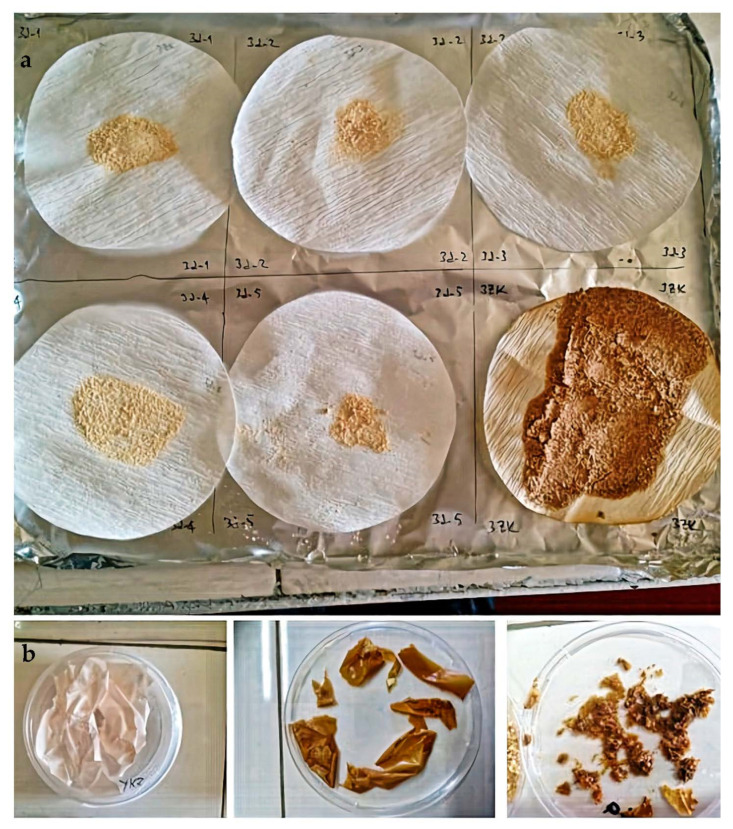
Images of eggshell membrane (**a**) from foam separation process; (**b**) before and after OLE adsorption in a batch experiment.

Images of the eggshell membrane before and after loading olive leaf extract are shown in Figure 9. As indicated in Table 2, the cases where different factors were applied together with OLE and ESM were investigated. These factors are clearly stated in Table 2. Figure 9 shows the effect of these factors on the obtained OLE-treated ESMs. With the change of factors, the change in the amount of OLE attached to the ESMs can be demonstrated visibly. As the amount of OLE bonded increases, the color of the eggshell membrane changes from white to a darker brown. As seen in Figure 9a, ESM processed together with OLE in the foam separation set-up at different process conditions had a color change from light to dark brown due to the adsorption of polyphenols present in the olive leaf extract. As a result of the adsorbed polyphenol amount on ESM in a batch experiment, the color change of ESM from white to dark brown can be observed in Figure 9b. This shows us that the efficiency of the OLE-loaded amount calculated is correct.

FTIR spectra of the eggshell membrane and OLE-loaded eggshell membrane are shown in Figure 3d. The existence of absorption bands in the FTIR spectrum of adsorbed polyphenols proved the accumulation of OLE on the ESM, since ESM was taken as background. The presence of peaks at certain adsorption bands in olive leaf extract and adsorbed polyphenols represented that the adsorption of polyphenols on ESM was accomplished.

The FTIR spectrum of ESM showed regions of amides A, B, I, II, and III were 3282, 2926, 1637, 1529, and 1236 cm^−1^, respectively. C-O stretching in phenols produced an absorption band in the 1300–1100 cm^−1^ region [32]. This band appeared at around 1240 cm^−1^ in the adsorbed polyphenols on ESM. Furthermore, the characteristic band of ESM at 1529 cm^−1^ shifted to 1534 cm^−1^, and the peak that appeared at 1637 cm^−1^ decreased after OLE polyphenol adsorption. Obtained results showed a shift in the aromatic amino acids due to their interaction with OLE polyphenols, which, it can be concluded, resulted from the interaction between polyphenols and proteins occurring at the molecular level. The broad strong peak between 3600 and 3200 cm^−1^ regions showed the O-H stretching. In the spectra, peaks representing the C-O stretching appeared at around 1075–1083, and 1000 cm^−1^. The intensities of the peaks in this region increased significantly. As a result of adsorbed polyphenols, the observed changes in the spectrum confirmed the polyphenol adsorption on ESM [32,35].

In the literature, Tang et al. (2003) also studied polyphenol interactions with both cellulose and collagen [36]. For the polyphenol interactions with cellulose and collagen, they concluded that the hydrophobic interactions are significantly important. The selective adsorption of the hydrolyzable tannins that are rich in gallolyl groups with collagen fiber was also reported in the literature [37]. Therefore, the adsorption of OLE polyphenols on ESM can be attributed to the hydrophobic interactions between the polyphenols and the collagen present in the structure of ESM.

**Figure 10:**
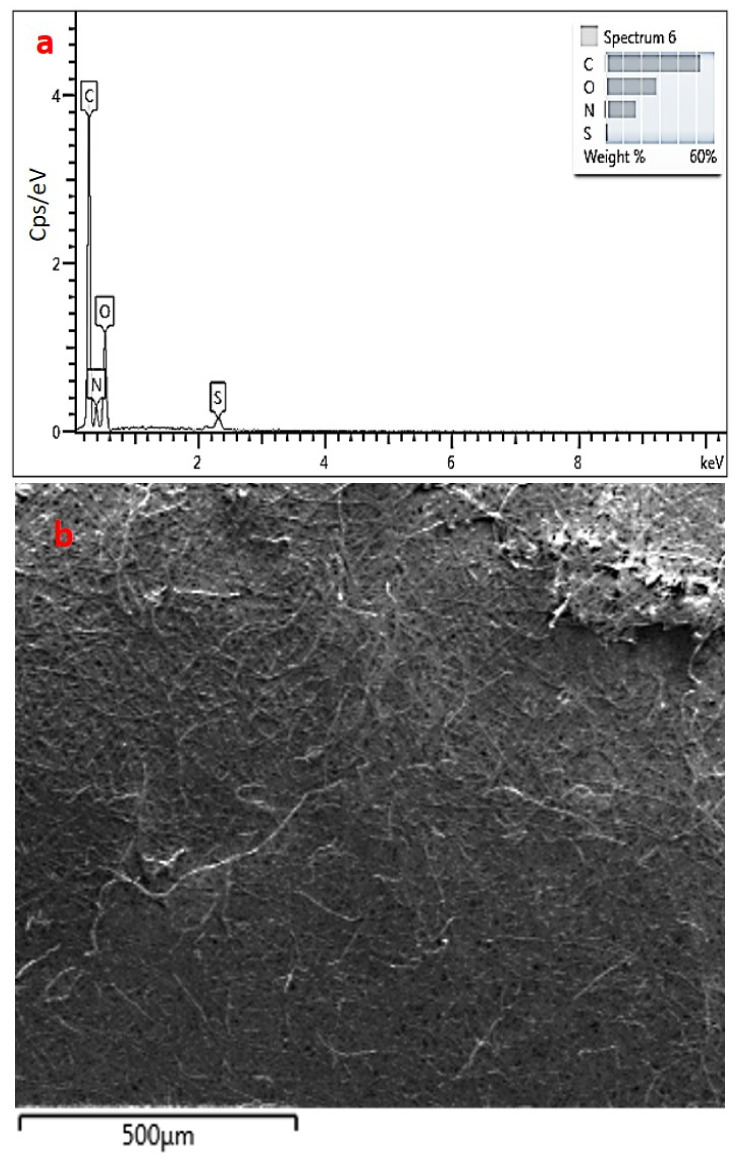
(**a**) EDS analysis of OLE-loaded eggshell membrane; (**b**) SEM image of the OLE-loaded eggshell membrane.

In Figure 10, EDS analysis of the OLE-loaded eggshell membrane is given.

As the SEM images revealed, the porosity between the fibrous structure of the eggshell membrane was coated with OLE. A smoother surface was observed, as seen in Figure 10b. The EDS analysis of the ESM loaded with OLE showed that the eggshell membrane consisted of C, N, O, and S, with 58.67, 16.55, 23.98, and 0.80 wt%, respectively. As seen from Figure 10a, with the adsorption of OLE to the eggshell membrane, carbon weight percent was increased by almost 12% compared to ESM without OLE.

### 3.4. Cytotoxic Effect of OLE Loaded Eggshell Membrane

**Figure 11:**
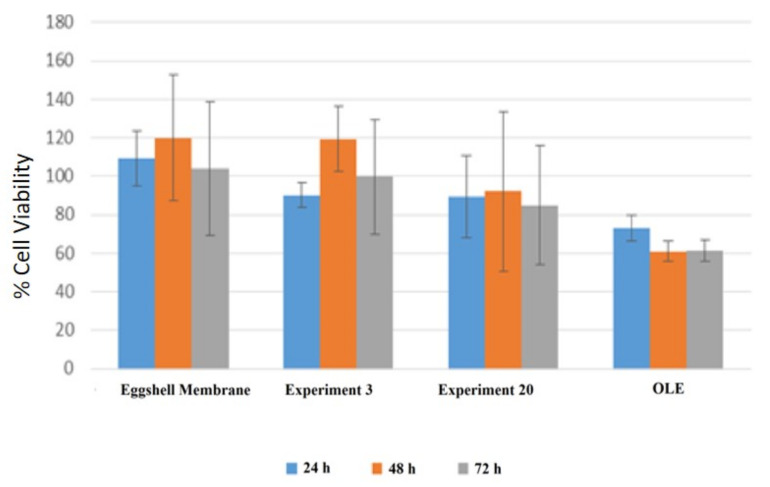
Cytotoxic activity of the OLE-loaded eggshell membrane on 3T3 cell.

In Figure 11, the results of cytotoxicity studies of the samples prepared with different process parameters for 24, 48, and 72 h are given.

The effect of OLE and OLE-loaded eggshell membrane on the viability of the 3T3 cells was investigated. In Figure 11, the incubation of OLE-loaded eggshells with cells at different time intervals is coded with colors. Blue bars represent 24 h incubation, brown bars represent 48 h incubation, and gray bars represent 72 h incubation. Besides, the margins of error in the calculated cell viability analysis are indicated as a line graph above the bars. The x-axis of the graph shows the eggshell, eggshell-OLE mixes prepared with the factors indicated in Table 2 (sample number 3 and 20), and pure OLE. The parameters used in the preparation of sample number 3 were the following: acetic acid concentration “0%”, eggshell size “2 mm”, time spent in column “1 h”, and average air flow velocity in column “0.1 cm/s”. The eggshell and membrane separation efficiency of sample number 3 is 38%. The parameters used in the preparation of sample number 20 were the following: acetic acid concentration “50%”, eggshell size “2 mm”, time spent in column “3 h”, and mean air flow velocity in column “0.3 cm/s”. The eggshell and membrane separation efficiency of sample number 3 is 47%. The Y-axis is the percentage expression of cell viability. The cytotoxic effect of OLE is observed in the graph. In addition, it has been shown that eggshell alone does not have any cytotoxic effect. One reason for this may be the difference in the amount of OLE loaded and the different release characteristics from the eggshell membrane. The OLE alone showed 60% cell viability, whereas the eggshell membrane showed 100% cell viability. The anti-cancer properties of the OLE could explain this result. As seen from Figure 11, 48-h inoculation showed the best products for all the samples.

The cell viability difference between ESM obtained from experiments 3 and 20 can be explained by the process conditions for separating the eggshell membrane. Even though similar efficiencies were obtained in both experiments, due to acetic acid present in the investigation. In addition, the different cytotoxic activities may depend on the loading conditions and amounts. The conditions in experiment 20 changed the adsorption properties of the eggshell membrane, which caused the 20% lower cell viability.

### 3.5. Antimicrobial Effect of OLE-Loaded Eggshell Membrane

**Figure 12:**
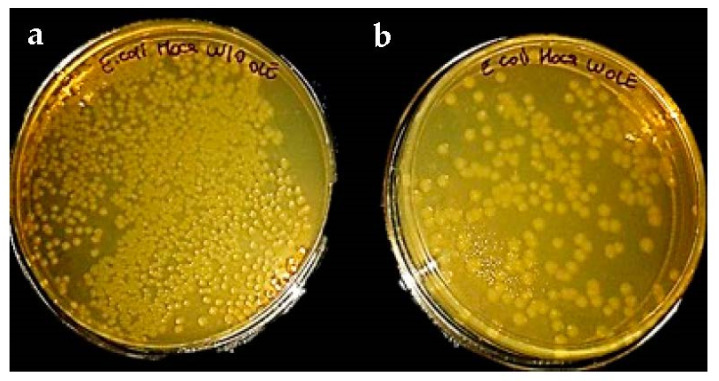
*E. coli* growth in the (**a**) eggshell membrane (**b**) OLE-loaded eggshell membrane.

In Figure 12, the antimicrobial effect of OLE-loaded eggshell membrane is shown in the agar surfaces. While microorganism growth in OLE-free eggshell membrane was not counted, 232 *E. coli* colonies were found in the sample of OLE-containing eggshell.

As seen from Figure 12, the presence of OLE drastically changed the *E.coli* growth in the agar medium. These results revealed that the antimicrobial properties of the OLE were preserved with the eggshell membrane.

Eggshell membrane can be considered as a source of typical type I collagen and may be utilized in various applications including functional food, cosmetic, biomedical, and pharmaceutical industries.

Although eggshell membrane has been shown to contain collagenous protein, eggshell membrane collagen has been proven to be of a very low autoimmune and allergic reaction. According to the result of research on a characteristic of the eggshell membrane and biosafety, the possibility of application in functional foods, cosmetics, and other industries is great [38].

Our results and others in the literature prove that adsorption is a powerful technique for removing or recovering polyphenols. Because of its low cost and regeneration possibility, it is also a suitable technique for industrial applications. If an edible biopolymer adsorbent such as ESM can be used in the adsorption of OLE polyphenols, then there will be no need for a desorption step, and therefore ESM will have antioxidative and antimicrobial properties for further use in industry, as a functional food or dietary supplement.

## 4. Conclusions

The present study suggested that ESM can be explored as an efficient and low-cost adsorbent for phenolic compounds. With this study, both olive leaf and ESM as agricultural and food processing wastes have been utilized. The FTIR, SEM, and EDS analyses showed changes in the eggshell membrane surface and the elemental composition after the OLE adsorption to the eggshell membranes. Cytotoxicity studies revealed that the OLE presence in the OLE-loaded eggshell membrane was influential on the 3T3 cells. By changing the process parameters, it was possible to improve the eggshell membrane’s adsorption properties, which improved the cytotoxic properties of the OLE-loaded membrane. By adding the phenolic compounds from the OLE, protein-based functional biomaterial with antioxidant and antimicrobial activities have successfully been produced. With the beneficial properties of ESM and OLE, it is possible to create an extensive range of applications for the cosmetic, functional food, dietary supplement, and pharmaceutical industries.

## Figures and Tables

**Table 1:** Independent variables and their levels used for Central Composite Design (CCD).

Independent Variable	Unit	Factor Symbol	Coded Levels	
			−1	1
Acetic acid concentration	%	A	0	100
Average eggshell size	mm	B	0.5	2
Treatment time in the column	h	C	1	5
Air flow rate	cm/s	D	0.1	0.5

**Table 2:** Central Composite Design (CCD) matrix and experimentally observed response.

Exp No	Acetic Acid Concentration % (*v/v*)	Average Eggshell Size (mm)	Time in the Column (h)	Flow Rate of the Air in the Column (cm/s)	Eggshell and Membrane Separation Efficiency (%)
1	0	0.5	1	0.1	45
2	100	0.5	1	0.1	66
3	0	2.0	1	0.1	38
4	100	2.0	1	0.1	68
5	0	0.5	5	0.1	42
6	100	0.5	5	0.1	77
7	0	2.0	5	0.1	36
8	100	2.0	5	0.1	65
9	0	0.5	1	0.5	40
10	100	0.5	1	0.5	69
11	0	5.0	1	0.5	38
12	100	5.0	1	0.5	71
13	0	0.5	5	0.5	42
14	100	0.5	5	0.5	70
15	0	2.0	5	0.5	38
16	100	2.0	5	0.5	81
17	0	1.25	3	0.3	34
18	100	1.25	3	0.3	74
19	50	0.5	3	0.3	43
20	50	2.0	3	0.3	47
21	50	1.25	1	0.3	45
22	50	1.25	5	0.3	56
23	50	1.25	3	0.1	44
24	50	1.25	3	0.5	40
25	50	1.25	3	0.3	47
26	50	1.25	3	0.3	34
27	50	1.25	3	0.3	45
28	50	1.25	3	0.3	46
29	50	1.25	3	0.3	47
30	50	1.25	3	0.3	38
31	50	1.25	3	0.3	40

## Data Availability

Data is contained within the article or Supplementary Material.

## Supplementary Materials

The following are available online at https://www.mdpi.com/article/10.3390/foods10040806/s1, Figure S1: Residual Plots for the Separation Efficency(a), Total Phenol Content(b) and Total Antioxidant Capacity(c), Table S1: Results of ANOVA for the prepared experimental design, Table S2: Independent variables and their levels used for CCD, Table S3: Response Surface Regression: Antioxidant Capacity versus L/S Ratio; Time (h), Table S4: Response Surface Regression: TPC versus L/S Ratio; Time (h).
