# Supplementary material for: Utilization of Eggshell Membrane and Olive Leaf Extract for the Preparation of Functional Materials
Source: Foods. 2021 Apr 9;10(4):806. doi: 10.3390/foods10040806 (PMC8069921; doi:10.3390/foods10040806)
Supplement: Supplementary file 1 [file foods-10-00806-s001.pdf]

Table S1 Results of ANOVA for the prepared experimental design

Term	Coef	SE Coef	T	P
Constant	52.5707	8.6646	6.067	0.000
A	−0.1656	0.1373	−1.206	0.0245
B	−6.2556	13.8297	−0.452	0.657
C*	−8.5237	4.7340	−1.801	0.091
D	19.4847	47.3404	0.412	0.686
A^2	0.0036	0.0012	3.065	0.007
B^2	0.2133	5.2906	0.040	0.968
C^2*	1.4050	0.7440	1.888	0.077
D^2	−72.0000	74.03997	−0.968	0.348
AB	0.0367	0.0320	1.147	0.268
AC	0.0137	0.0120	1.147	0.268
AD	0.1125	0.1199	0.939	0.362
BC	−0.2500	0.7990	−0.313	0.758
BD	12.5000	7.9903	1.564	0.137
CD	1.5625	2.9964	0.521	0.609

S = 4.79417 PRESS = 1418.39; R^2=0.9408 R-Sq(pred) = 77.18% R-Sq(adj) = 88.91%;***R^2=0.92**

Table S2 Independent variables and their levels used for CCD

Independent Variables	Unit	Factors Symbol	Coded Levels	
			−1	1
Liquid/Solid Ratio(L/S)	-	X1	10	30
Treatment Time in the Column	hour	X2	6	24
Ethanol Concentration	%	X3	0	80

Table S3. Response Surface Regression: Antioxidant Capacity versus L/S Ratio; Time (hr);

Estimated Regression Coefficients for Antioxidant capacity

Term	Coef	SE Coef	T	P
Constant	9312.70	2649.11	3.515	0.006
X_1	−93.02	274.31	−0.339	0.742
X_2	490.16	218.79	2.240	0.049
X_3	−119.77	48.16	−2.487	0.032
X_1^2	-0.17	6.54	−0.025	0.980
X_2^2	−21.51	6.54	−3.288	0.008
X_3^2	1.93	0.41	4.716	0.001
$X_1.X_2$	4.29	3.84	1.120	0.289
$X_1.X_3$	1.48	0.96	1.543	0.154
$X_2.X_3$	1.21	0.96	1.265	0.235

S = 1084,76 PRESS = 85852664

R-Sq = 95.85% R-Sq(pred) = 69.75% R-Sq(adj) = 92.12%

Analysis of Variance for Antioxidant

Source	DF	Seq SS	Adj SS	Adj MS	F	P
Regression	9	272,047,514	272,047,514	30,227,502	25.69	0.000
Linear	3	235,079,966	11,031,132	3,677,044	3.12	0.075
Square	3	30,809,714	30,809,714	10,269,905	8.73	0.004
Interaction	3	6,157,834	61,57,834	2,052,611	1.74	0.221
Residual Error	10	11,767,102	11,767,102	1,176,710		
Lack-of-Fit	5	11,085,380	11,085,380	2,217,076	16.26	0.004
Pure Error	5	681,722	681,722	136,344		
Total	19	283,814,616				

Unusual Observations for Antioxidant

Obs	StdOrder	Antioxidant	Fit	SE Fit	Residual	St Resid
16	14	22474.000	20324.618	760.038	2149.382	2.78 R

R denotes an observation with a large standardized residual.

Predicted Response for New Design Points Using Model for Antioxidant

Point	Fit	SE Fit	95% CI	95% PI
1	9697.3	966.098	(7544.7; 11849.9)	(6460.7; 12933.9)
2	10342.0	760.038	(8648.5; 12035.5)	(7390.8; 13293.2)
3	10171.6	760.038	(8478.1; 11865.1)	(7220.4; 13122.8)
4	10660.0	760.038	(8966.5; 12353.5)	(7708.8; 13611.2)
5	8428.9	966.098	(6276.3; 10581.5)	(5192.3; 11665.5)
6	12407.5	372.915	(11576.5; 13238.4)	(9851.6; 14963.3)
7	19965.1	966.098	(17812.5; 22117.7)	(16728.5; 23201.7)
8	17149.5	966.098	(14996.9; 19302.1)	(13912.9; 20386.1)
9	12407.5	372.915	(11576.5; 13238.4)	(9851.6; 14963.3)
10	17208.4	966.098	(15055.8; 19361.0)	(13971.8; 20445.0)
11	12407.5	372.915	(11576.5; 13238.4)	(9851.6; 14963.3)

12	12407.5	372.915	(11576.5; 13238.4)	(9851.6; 14963.3)
13	12407.5	372.915	(11576.5; 13238.4)	(9851.6; 14963.3)
14	7697.9	966.098	(5545.3; 9850.5)	(4461.3; 10934.5)
15	12777.6	760.038	(11084.1; 14471.1)	(9826.4; 15728.8)
16	20324.6	760.038	(18631.1; 22018.1)	(17373.4; 23275.8)
17	8147.0	966.098	(5994.4; 10299.6)	(4910.4; 11383.6)
18	12407.5	372.915	(11576.5; 13238.4)	(9851.6; 14963.3)
19	18306.5	966.098	(16153.9; 20459.1)	(15069.9; 21543.1)
20	12004.0	760.038	(10310.5; 13697.5)	(9052.8; 14955.2)

Analysis of Variance for Antioxidant Capacity (R^2=0.9585)

Source	DF	Seq SS	Adj SS	Adj MS	F	P
Regression	9	272,047,514	27,2047,514	30,227,502	25.69	0.000
Linear	3	235,079,966	11,031,132	3,677,044	3.12	0.075
Square	3	30,809,714	30,809,714	10,269,905	8.73	0.004
Interaction	3	6,157,834	6157834	2,052,611	1.74	0.221
Residual Error	10	11,767,102	11,767,102	1,176,710		
Lack-of-Fit	5	11,085,380	11,085,380	2,217,076	16.26	0.004
Pure Error	5	681,722	681,722	136,344		
Total	19	28,3814,616				

Table S4. Response Surface Regression: TPC versus L/S Ratio; Time (hr);

Estimated Regression Coefficients for Total Phenol Content

Term	Coef	SE Coef	T	P
Constant	0.105738	0.047151	2.243	0.049
X_1	−0.002571	0.004882	−0.527	0.610
X_2	0.002010	0.003894	0.516	0.617
X_3	0.002304	0.000857	2.688	0.023
X_1^2	0.000077	0.000116	0.660	0.524
X_2^2	−0.000123	0.000116	−1.058	0.315
X_3^2	−0.000019	0.000007	−2.604	0.026
$X_1.X_2$	0.000019	0.000068	0.275	0.789
$X_1.X_3$	−0.000004	0.000017	−0.238	0.817
$X_2.X_3$	−0.000002	0.000017	−0.128	0.901

S = 0.0193076 PRESS = 0.0139841

R-Sq = 72.29% R-Sq(pred) = 0.00% R-Sq(adj) = 47.35%

Analysis of Variance for Total Pheenol Content

Source	DF	Seq SS	Adj SS	Adj MS	F	P
Regression	9	0.009726	0.009726	0.001081	2.90	0.056
Linear	3	0.003745	0.003262	0.001087	2.92	0.087
Square	3	0.005925	0.005925	0.001975	5.30	0.019
Interaction	3	0.000055	0.000055	0.000018	0.05	0.985
Residual Error 10		0.003728	0.003728	0.000373		
Lack-of-Fit	5	0.000754	0.000754	0.000151	0.25	0.921
Pure Error	5	0.002974	0.002974	0.000595		
Total	19	0.013454				

Unusual Observations for Total Phenol Content

Obs	StdOrder	Total phenol	Fit	SE Fit	Residual	St Resid
13	17	0.109	0.155	0.007	−0.046	−2.55 R

R denotes an observation with a large standardized residual.

Predicted Response for New Design Points Using Model for Total Phenol Content

Point	Fit	SE Fit	95% CI	95% PI
1	0.116248	0.0171955	(0.077934; 0.154562)	(0.058640; 0.173856)
2	0.157109	0.0135279	(0.126967; 0.187251)	(0.104580; 0.209638)
3	0.128709	0.0135279	(0.098567; 0.158851)	(0.076180; 0.181238)
4	0.113109	0.0135279	(0.082967; 0.143251)	(0.060580; 0.165638)
5	0.127348	0.0171955	(0.089034; 0.165662)	(0.069740; 0.184956)
6	0.155227	0.0066375	(0.140438; 0.170017)	(0.109736; 0.200718)
7	0.123048	0.0171955	(0.084734; 0.161362)	(0.065440; 0.180656)
8	0.110948	0.0171955	(0.072634; 0.149262)	(0.053340; 0.168556)
9	0.155227	0.0066375	(0.140438; 0.170017)	(0.109736; 0.200718)
10	0.144848	0.0171955	(0.106534; 0.183162)	(0.087240; 0.202456)
11	0.155227	0.0066375	(0.140438; 0.170017)	(0.109736; 0.200718)
12	0.155227	0.0066375	(0.140438; 0.170017)	(0.109736; 0.200718)

13	0.155227	0.0066375	(0.140438; 0.170017)	(0.109736; 0.200718)
14	0.085848	0.0171955	(0.047534; 0.124162)	(0.028240; 0.143456)
15	0.168709	0.0135279	(0.138567; 0.198851)	(0.116180; 0.221238)
16	0.136709	0.0135279	(0.106567; 0.166851)	(0.084180; 0.189238)
17	0.104448	0.0171955	(0.066134; 0.142762)	(0.046840; 0.162056)
18	0.155227	0.0066375	(0.140438; 0.170017)	(0.109736; 0.200718)
19	0.149448	0.0171955	(0.111134; 0.187762)	(0.091840; 0.207056)
20	0.157109	0.0135279	(0.126967; 0.187251)	(0.104580; 0.209638)

Analysis of Variance for Total Phenol Content (R^2=0.7229)

**Source**	**DF**	**Seq SS**	**Adj SS**	**Adj MS**	**F**	**P**
Regression	9	0.009726	0.009726	0.001081	2.90	0.056
Linear	3	0.003745	0.003262	0.001087	2.92	0.087
Square	3	0.005925	0.005925	0.001975	5.30	0.019
Interaction	3	0.000055	0.000055	0.000018	0.05	0.985
Residual Error	10	0.003728	0.003728	0.000373		
Lack-of-Fit	5	0.000754	0.000754	0.000151	0.25	0.921
Pure Error	5	0.002974	0.002974	0.000595		
Total	19	0.013454				

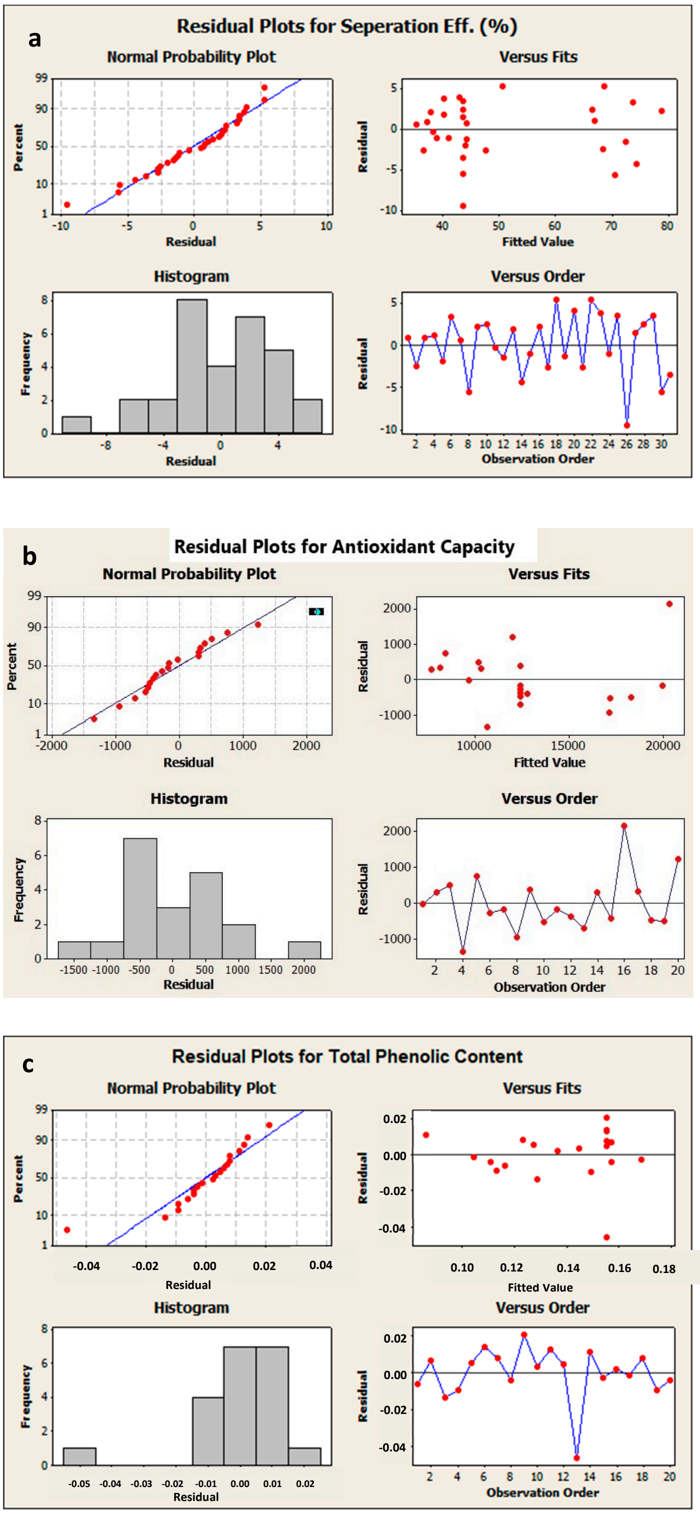
a
Residual Plots for Seperation Eff. (%)
Normal Probability Plot
Percent
Residual
Versus Fits
Residual
Fitted Value
Histogram
Frequency
Residual
Versus Order
Residual
Observation Order
b
Residual Plots for Antioxidant Capacity
Normal Probability Plot
Percent
Residual
Versus Fits
Residual
Fitted Value
Histogram
Frequency
Residual
Versus Order
Residual
Observation Order
c
Residual Plots for Total Phenolic Content
Normal Probability Plot
Percent
Residual
Versus Fits
Residual
Fitted Value
Histogram
Frequency
Residual
Versus Order
Residual
Observation Order

Figure S1 Residual Plots for the Separation Efficency(a).Total Phenol Content(b) and Total Antioxidant Capacity(c)
